# CB2 Agonist (AM1241) Improving Effect on Ovalbumin-Induced Asthma in Rats

**DOI:** 10.22037/IJPR.2020.15456.13104

**Published:** 2020

**Authors:** Ali Parlar, Seyfullah Oktay Arslan

**Affiliations:** a *University of Adiyaman, Faculty of Medicine, Department of Pharmacology, Adiyaman, Turkey. *; b *Pharmacology Department, Faculty of Medicine, Yildirim Beyazit University, Ankara, Turkey*

**Keywords:** CB2 receptor, Ovalbumin, Asthma, CB2 agonist, AM1241

## Abstract

Asthma is a disease characterized by spontaneous contraction of the airways in response to a wide variety of endogenous and exogenous stimuli. Many asthma models are used to mimic the human asthma model in the literature. In order to better understand the role of the cannabinoid (CB) 2 receptor in the ovalbumin (OVA)-induced asthma model, a combination of both selective CB2 agonist (AM1241) and antagonist (AM630) was used to improve inflammatory hypersensitivity and edema in rats. In the present study, it was found that OVA decreased body weight (*p* < 0.05), increased lung weights (*p *< 0.05), increased diastolic and systolic blood pressure (*p *< 0.001), and caused irregularity in pulmonary functions (*p *< 0.001). Moreover, CB2 agonist was found not to reduce body weight, cause blood pressure and respiratory irregularities (*p *< 0.05). OVA led to increase in IgE, TNF-α, IL-4, MDA level (*p *< 0.001), and total WBC count (*p *< .05). CB2 treatment caused to reduce the number of total WBC and the level of total protein in BALF, to hinder to increase level of MDA, IgE, TNF-α, and IL-4 (*p *< 0.05) in BALF or serum or lung tissue. But CB2-antagonist treatment prevented the protective effect of CB2 agonist. The aim of this study was to study the role of the CB2 receptor in the OVA induced asthma model, to improve inflammatory hypersensitivity, and edema in the rats. The results suggested that CB2 agonist administration to OVA induced asthmatic rats via anti-asthmatic potential through inhibition of parameters such as IgE, IL-4, TNF-α, microvascular escape, and oxidative stress.

## Introduction

Asthma is a disease characterized by spontaneous contraction of the airways in response to a wide variety of endogenous and exogenous stimuli, affecting approximately 300 million people worldwide and about 20% of the population in developed countries. (1, 2). Various cytokines and inflammatory cells such as mast cells, T cells, and neutrophils eosinophils in the hypersensitivity of airways are involved (3–5). Moreover, with the increase in mucus secretion of the airways, contraction of smooth muscles and edema are also observed. Shortness of breath, wheezing, coughing and intermittent breathing are among the symptoms of asthma. Epithelial fibrosis is seen in the pathology of asthma, hyperplasia, and also metaplasia in goblet cells, excessive mucus secretion, hyperplasia and hypertrophy of airway smooth muscle are observed (6). 

Animal models of asthma are used to better understand the pathophysiological mechanism of asthma due to many causes and difficulty of human ethics committee. For this purpose, many animal species and many asthma models are used to mimic the human asthma model in the literature. Among these models, many antigens such as fruit fly Drosophila melanogaster, gene-knockout mice model, and OVA are used to make asthma. OVA is an antigen used in both acute and chronic asthma models because it forms an immunological and non-immunologic asthma model in the rats (1). Acute asthma is characterized by total immunoglobulin in serum and specific IgE increase, airway inflammation, epithelial hypertrophy, hyperplasia in goblet cells, airway hyperresponsiveness, elevated serum cytokine levels and inflammatory cell migration to the region. In this context, in order to treat asthma, many agents especially anti-inflammatory agents were used and will continue to be used (1, 2, 7, 8). Cannabinoid, delta(Δ)9-tetrahydrocannabinol (THC), is a psychoactive substance derived from marijuana (*Cannabis sativa*) (9). Since the discovery of cannabinoid 1 and cannabinoid 2 receptors in the body (CB1, CB2, respectively) in 1992 by Johnson et al., many studies have been carried out, especially anti-inflammatory properties (10–14). The first defined receptor is the CB1 receptor. They are specific, high affinity cannabinoid binding sites found in both the central nervous system (CNS) and peripheral tissues. They are especially found in the most intense ratios in the areas responsible for movement control, sensory perception, consciousness, and short-term memory (cerebral cortex and hippocampus), and in areas responsible for motor function and movement (basal ganglia and cerebellum) (15). It was determined that the CB2 receptor was found only peripheral tissue such as blood cells and the immune system (especially B cells, mast cells, T4-T8 cells, natural killer cells and macrophages). Activation of the CB2 receptor through the Gi/o protein coupled inhibits adenylate cyclase and eventually activates some kind of protein kinase (10, 16–20).

In order to better understand the role of the CB2 receptor in the OVA-induced asthma model, a combination of both selective CB2 agonist (AM1241, (R, S) -3- (2-Iodo-5-nitrobenzoyl) -1- (1-methyl-2-piperidinylmethyl) -1) and antagonist (AM630, 6-Iodo-2-methyl-1- [2- (4-morpholinyl) ethyl] -1H-indol-3-yl] (4-methoxyphenyl) methanone, Iodopravadoline) was used to improve inflammatory hypersensitivity and edema in rats (21).

## Experimental

*Chemicals*

Aluminium hydroxide (Al(OH)_3_) (Alum), ovalbumin, AM1241, AM630, dimethyl sulfoxide (DMSO), glacial acetic acid, gentian violet solution, giemsa stain, trichloroacetic acid (TCA), thiobarbituric acid (TBA), Na_2_HPO_4_, 5,5′-Dithiobis(2-nitrobenzoic acid) (DTBN), and sodium citrate were purchased from Sigma-Aldrich  (St. Louis, MO, USA).

*Animals*

Male Wistar rats weighing 180-250 g were used in this study. The animals were provided from the Experimental Research Center of Adiyaman University. The ethical permission for the study were taken from the Adiyaman University Animal Experiments local ethics committee (Ethics Committee decision no. 2018/15) and the study was conducted in Adiyaman University. Animals housed at 22±1 °C under 12:12 h light-dark cycle. The animals were allowed free access to standard laboratory chow and water. All procedures complied with the standards for the care and use of animals as stated in Guide for the Care and Use of Laboratory Animals. Before experiment, in each group, six rats were randomly divided into 5 groups. 

*Experimental Design*

*Animal Sensitization and Challenged*

**Figure 1:**
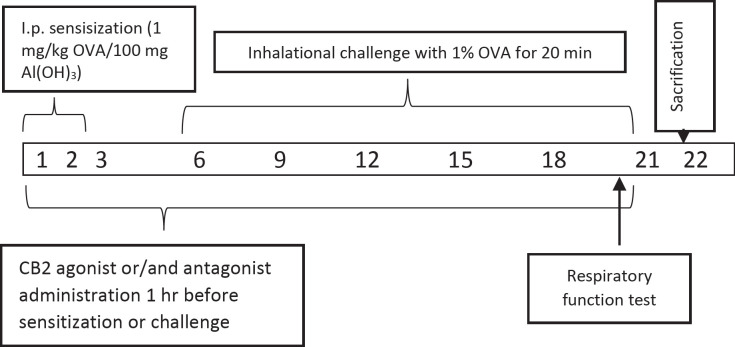
Schematic diagram of experimental design. Asthma was induced by ip. administration of 1 mg/kg OVA/100 mg aluminium hydroxide (Al(OH)_3_) suspended in 1 ml saline for 3 consecutive days then inhalation of 1% OVA with all body nebulizer at day 6, 9, 12, 15, 18, and 21

In previous studies the rats were used successfully to set the allergic airway inflammation model (22–24). Briefly, all rats in the group except saline control group were given 100 mg of Alum in 0.9% sterile saline with 1 mg / kg of OVA per day intraperitoneally for 3 days (23). Alum was given as adjuvant due to boost the immune response to produce more antibodies and long-lasting immunity. All animals in groups from, but saline control group, the 6th, 9th, 12th, 15th, 18th, and 21th days of the experiment were challenged to inhalation with 1% OVA whole-body nebulizer for 20 min a day for 0.8 m^3^ (n = 6). All the animals were sacrificed on day 22 (Figure 1). Doses of CB2 agonists and antagonists have been referenced from the previous studies (25).

*Experimental groups*

Experimental groups are Group 1. Saline Control, Group 2. Ovalbumin (OVA), Group 3. Ovalbumin + CB2 agonist (OVAA), Group 4. Ovalbumin + CB2 antagonist + agonist (OVAA+A) and Group 5. DMSO (2% v/v in saline) (OVA + Vehicle).

*Measurement of Pulmonary Function Tests*

Pulmonary function tests (PFT) were recorded on the 22st day of the experiment using whole body plethysmography (Emka Technologies, Paris, France) as in the previous studies (26–28). In the present study, Peak inspiratory flow (PIF), Peak expiratory flow (PEF), expiratory volume (EV), tidal volume (TV), the frequency of breathing (f), and enhanced pause (P_enh_) value were measured. The rats were allowed to enter the room for 15 min without recording. The recordings were performed for 10 min to form the baseline values.

*Determination of RAT weights*

The Weights of the animals which are in group of 22^th^ day were recorded by being measured periodically for all application groups during the experiment. The ratio of lung weight to body weight was calculated according to formula (lung/body weight) * 100.

*Measurement of systolic and diastolic blood pressure*

Systolic (S) and diastolic (D) blood pressures of the animals which are in group of 22^th^ day was recorded by being measured for all application groups during the experiment by using Noninvasive Blood Pressure Measurement System (May, NIBP250).

*Collection of bronchoalveolar lavage fluid and its determination*

On the 22^th^ day after 24 h of the last treatment, the rats were anesthetized with ketamine (100 mg kg-1, I.P) and harvested 3 ml blood samples from vena jugularis and sacrificed. Thorax was opened by a median incision and trachea was cannulated with a plastic catheter attached to 10 mL syringe. After bronchoalveolar lavage (BALF) was performed with a total volume 25 mL sterile saline in 5 mL portions, with gentle massage of lungs they were combined. To determine total leukocyte count, 1 mL of BALF was stained by Turk solution (1 mL glacial acetic acid, 1 mL gentian violet solution 1% and 100 mL pure water) and total White blood cells (WBC) were determined in duplicate using a hemocytometer (22, 29). The rest of BALF centrifuged at 300 g for 10 min at 4 °C to obtain the supernatant for oxidant studies (7)  and cells were collected for cell counts from cell pellet. The cell samples from the cell pellet were diluted with 200 µL saline, followed by rubbing onto a 20 µL sterile slide and allowed to dry. The cells were stained with Giemsa to analyze cellular differentiation of macrophages, neutrophils, and lymphocytes. To determine the ratio of cells to WBC, 100 cells were counted under light microscopy and cell types were determined as percentage (30). 

*Preparation of lung tissue*

The lungs were gently separated from the bronchi and the lungs were weighed, washed with cold saline and homogenized in isotonic saline. The homogenates were transferred to tubes and stored at -20 °C until the experiments were performed.

*Determination of Total Protein*

ELISA procedures were used to determine marker levels as biochemical. Piercetm BCA Protein Assay Kit (Thermo Scientific) was used for the determination of total protein in tissue and BALF.

*Determination of IL-4, TNF-α, and IgE levels*

IL-4, TNF-α and IgE were measured in lung homogenate, serum and BALF using ELISA kits for rat (Abcam ab100770, ab46070 and ab157736, Istanbul/Turkey, respectively) and this process was performed 2 times, as described by the manufacturer (31, 32) and the results expressed as pg/g wet tissue, pg/mL of serum or BALF. 

*Evaluation of MDA *content

Malondialdehyde (MDA) measurements in lung tissue, serum and BALF. The lipid peroxide levels, as MDA concentrations, in the lung tissues were according to the method previously described (33). Briefly the tissue samples were homogenized in an ice bath, ice-cold TCA by adding 10 mL of 10% TCA per g of tissue, with an ultrasonic tissue homogenizer. After two consecutive centrifugations at 3,000 g for 15 min, 500 µL supernatant was mixed with equal volume of 0.67% TBA and heated to 100 °C for 15 min. The absorbance of the samples was then measured by using spectrophotometry at 535 nm. Each assay was performed in duplicate.

*Determination of GSH level*

Glutathione (GSH) levels in lung tissue, serum and BALF were measured by a modified Elman method (34). Briefly, lung tissues were homogenized in 10 mL TCA which is at the rate of 10%. It was then centrifuged at + 4 °C for 15 min. Afterwards, 0.5 ml of supernatant was taken, and mixed with 0.3 M 2 mL Na_2_HPO_4_. The mixture was thoroughly vortexed. This mixture was vortexed by the addition of 0.2 ml DTBN (prepared by dissolving in 1% sodium citrate). Finally, it was measured at 412 nm by using spectrophotometry.

*Statistical analyses*

Average values of ventilatory variables were obtained on a minute by minute basis using data analysis software (DataAnalyst; EMKA). All values are reported as mean ± standard deviation. Statistical analysis was performed using one-way analysis of ANOVA. Statistical significance was set at *p*< 0.05. Data analysis was performed by using GraphPad Prism 7.0 software (GraphPad, San Diego,USA).

## Results

*Effect of CB2 agonist treatment on lung function tests*

**Figure 2.**
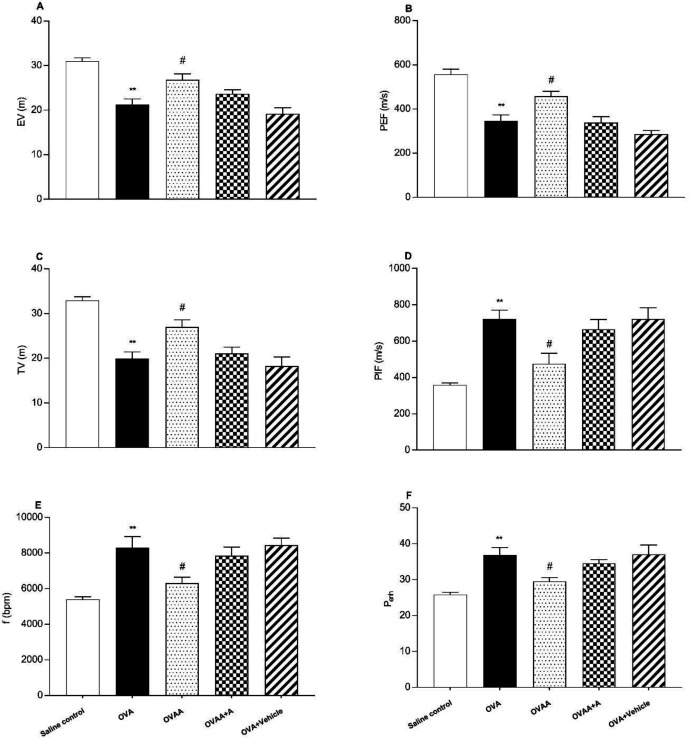
Effect of CB2 agonist on OVA-induced asthma in expiratory volume (A), Peak expiratory flow (B), tidal volume (C), Peak inspiratory flow (D), the frequency of breathing (E) and enhanced pause (F). Data are expressed as mean ± S.E.M. (n = 6) and one-way ANOVA followed by Tukey’s multiple range test. **p < 0.001 as compared to saline control group, #*p* < 0.05 as compared to OVA group

Non-invasive pulmonary function test (PFT) was used to determine the severity of lung injury. Peak expiratory flow, tidal volume and expiratory volume decreased significantly in OVA group when OVA group and Saline control group were compared (*p*<0.001). Peak inspiratory flow (PIF), respiratory frequency (f) and enhanced pause (Penh) also increased significantly in the OVA group compared to the saline control group (*p *<0.001). When OVAA group and OVA group were compared, peak expiratory flow (PEF) (*p* = 0.0176), Tidal volume (TV) (*p* = 0.02), and expiratory volume (*p*=0.0161) were significantly increased in OVAA group. Peak inspiratory flow (PIF) (*p*=0.0152), respiratory frequency (f) (*p*=0.0226) and enhanced pause (Penh) (*p*=0.0314) were also significantly decreased in the OVAA group compared to the OVA group (Figure 2). 

*Effect of CB2 agonist treatment on OVA-Induced Asthma in body, lung and lung/body weight ratio, systolic and diastolic blood pressure*

**Figure 3.**
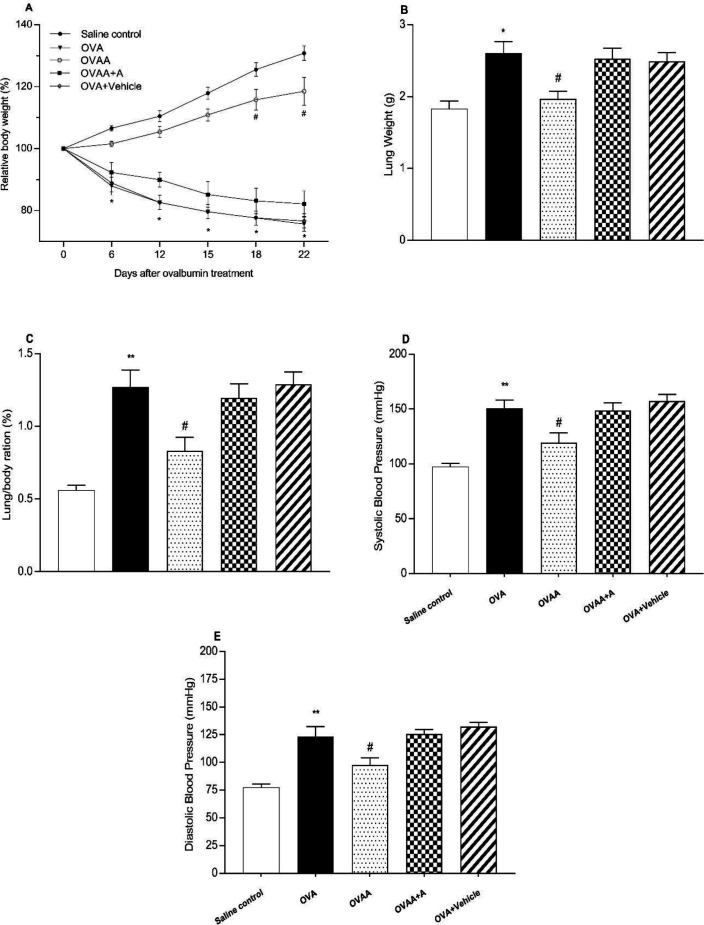
Effect of CB2 agonist on OVA-induced asthma in body(A) and lungs weight(B), lung/body ratio (C), systolic(D) and diastolic(E) blood pressure of rats. Data are expressed as mean ± S.E.M. (n = 6) and one-way ANOVA followed by Tukey’s multiple range test. ^*^p <0.05 or ^**^p < 0.001 as compared to saline control group,^ #^*p *< 0.05 as compared to OVA group

In the present study, OVA-induced asthma was found to be closely related to weight loss and increased lung weight. When the OVA group was compared with the Saline Control Group, the body weight of the OVA group was significantly reduced (*p *<0.001); however, lung weight (*p*=0.0024), and systolic (*p*<0.001) and diastolic (*p*<0.001) blood pressures were found to be increased. When OVAA and OVA groups were compared, the relative body weight of OVAA group increased significantly (*p*<0.001) and the relative lung weight (*p*=0.0155), systolic (*p *= 0.0207) and diastolic (*p *= 0.0311) blood pressures were significantly reduced by CB2 agonist (Figure 3).

*Effect of CB2 agonist treatment on cell counts*

**Figure 4.**
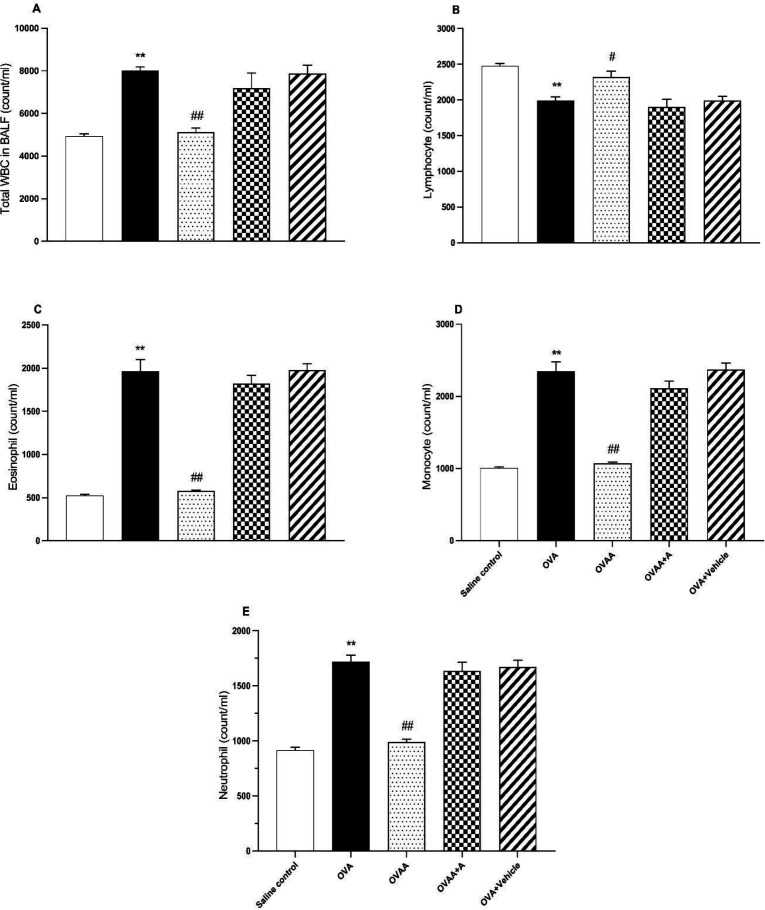
Effect of CB2 agonist on OVA-induced asthma in count of total WBC (A), lymphocyte (B), eosinophil (C), monocyte (D), and neutrophil (E) in BALF. Data are expressed as mean ± S.E.M. (n = 6) and one-way ANOVA followed by TUKEY’s multiple range test. ^**^p < 0.001 as compared to saline control group,^ #^*p *< 0.05 or ^##^*p *< 0.001 as compared to OVA group

In BALF, when OVA group and saline control groups were compared in terms of number of cells, it was observed that lymphocyte counts decreased significantly in OVA group but total WBC and other cell counts increased significantly (*p *<0.001). While there was no statistically significant difference between the OVAA group and the saline control groups in terms of cell numbers (*p*=0.9041), it was observed that the number of lymphocytes increased significantly (*p *=0.0317), total WBC and other cell numbers decreased compared to the OVA group (Figure 4). 

As seen in Table 1, total WBC count, percentages of eosinophils, monocytes, and neutrophils in BALF of OVA group were significantly increased but percentage of lymphocytes decreased compared to the control group. Total WBC count, percentage of eosinophil, monocyte and neutrophil in BALF of OVAA group were significantly decreased, but percentage of lymphocyte was increased compared to the OVA group (Table 1).

**Figure 5:**
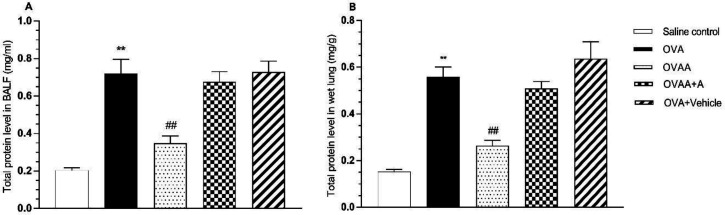
Effect of CB2 agonist on OVA-induced asthma in total protein in BALF (A) and lung (B) of rats. Data are expressed as mean ± S.E.M. (n = 6) and one-way ANOVA followed by Tukey’s multiple range test. ^**^p <0.001 as compared to saline control group,^ ##^*p *< 0.001 as compared to OVA group

The amount of total protein in the lung tissue and BALF is among the important determinants of the extent of damage in the asthmatic lung. Therefore, we investigated the effect of CB2 agonist by measuring total protein content in lung and BALF. In this context, when the OVA group and saline control groups were compared in terms of total protein content, the increase of total protein content in OVA groups was found to be statistically significant (*p* < 0.001). When OVAA group and OVA groups were compared in terms of total protein content, total protein decrease in the OVAA group was found to be statistically significant (*p *< 0.001) (Figure 5).

*Effect of CB2 agonist treatment on IL-4, and TNF-α levels*

**Figure 6:**
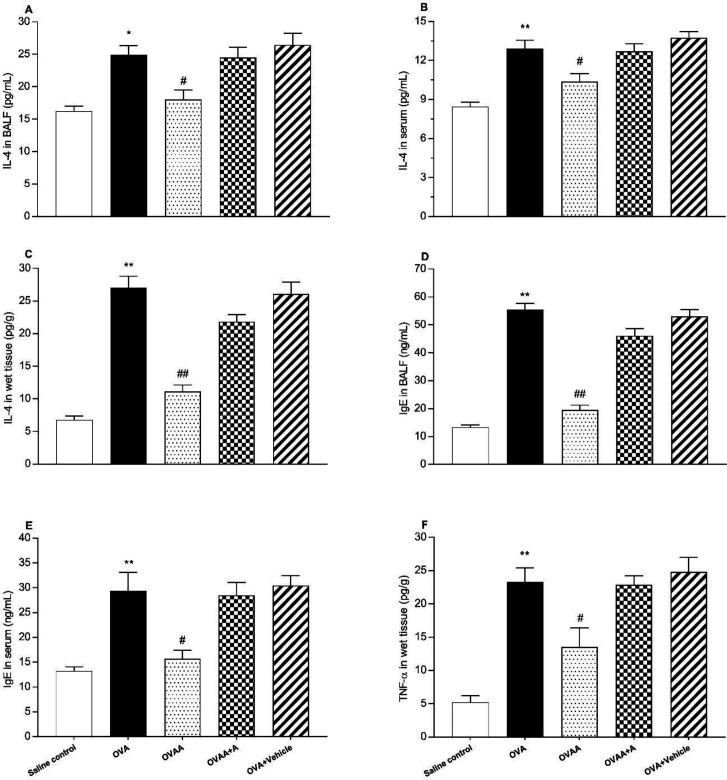
Effect of CB2 agonist on OVA-induced asthma in IL-4 level in BALF (A), in IL-4 level in serum (B), in IL-4 level in lung tissue (C), in IgE level in BALF (D), in IgE level in serum (E), TNF-α level in lung tissue (F). Data are expressed as mean ± S.E.M. (n = 6) and one-way ANOVA followed by Tukey’s multiple range test. ^*^p < 0.05 or ^**^p < 0.001 as compared to saline control group,^ #^*p *< 0.05 or ^##^*p *< 0.001 as compared to OVA group

The other criteria for determining the extent of lung injury are the IL-4 level in the lung tissue in the BALF and the serum, the level of IgE in the serum and in the BALF, and the TNF- α levels in the lung tissue. When the OVA group and saline control groups were compared in terms of IL-4 in BALF (*p *= 0.0021), serum and tissue (*p* < 0.001), IgE in BALF, serum and tissue (*p* < 0.001), and TNF-α in tissue (*p *< 0.001) levels, the increase in all of these parameters in OVA groups was statistically significant. IL-4 in the BALF, serum and tissue (*p *= 0.0178, *p*=0.0268, *p *< 0.001), IgE in the BALF and serum (*p *< 0.001, *p *= 0.0038), and TNF-α in the tissue (*p *= 0.0163) were found significantly lower in OVAA group compared to OVA group (Figure 6).

*Effect of CB2 agonist treatment on MDA, and GSH levels*

**Figure 7:**
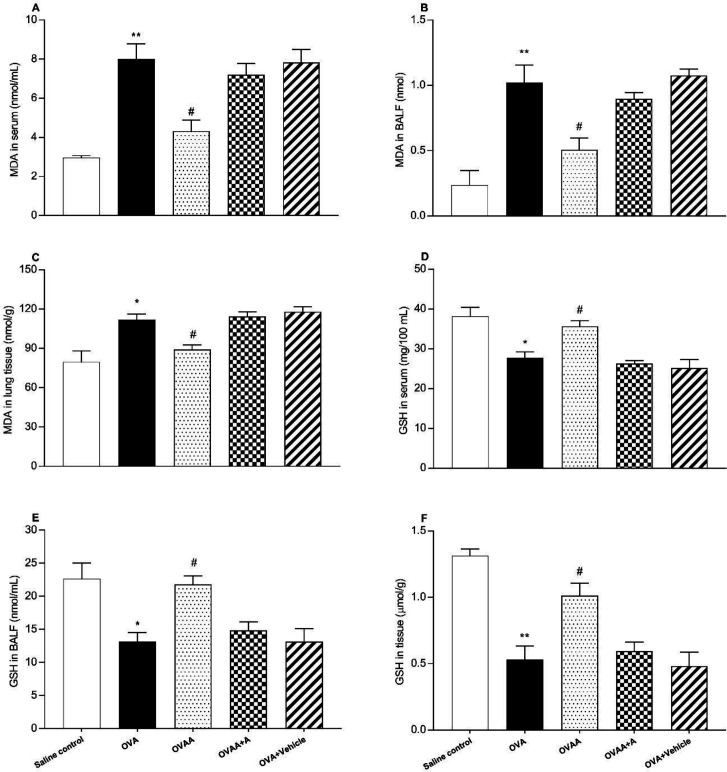
Effect of CB2 agonist on OVA-induced asthma in MDA content in serum (A), in BALF (B), in lung tissue (C), GSH level in serum (D), in BALF (E) and in tissue (F). Data are expressed as mean ± S.E.M. (n = 6) and one-way ANOVA followed by Tukey’s multiple range test. ^*^p < 0.05 or ^**^p < 0.001 as compared to saline control group,^ #^*p *< 0.05 as compared to OVA group

The other criteria for determining the extent of lung injury are the MDA content and GSH level in the lung tissue, in the BALF and in the serum. In this context, OVA and saline control groups in terms of MDA level in lung tissue (*p* = 0.0016), serum (*p *< 0.001) and BALF (*p *< 0.001) were found to be significantly increased in the OVA groups. When OVAA group and OVA groups were compared in terms of MDA level, the decrease in MDA level in serum (*p* = 0.0013), BALF (*p* = 0.0054) and tissue (*p* = 0.0369) was found statistically significant in OVAA group (Figure 7A, B, C). 

When the OVA group and Saline control group were compared in terms of GSH level, its level was found to be significantly decreased in the serum (*p* = 0.002), BALF (*p* = 0.0047) and tissue (*p* < 0.001). When the OVAA group and OVA group were compared in terms of GSH level, its level was found to be significantly increased in the serum (*p* = 0.0226), BALF (*p* = 0.0109) and tissue (*p* = 0.0046) (Figure 7D, E, F).

**Table 1:** Effect of CB2 agonist on OVA-induced asthma in differential cell counts in BALF of rats. Data are expressed as mean ± S.E.M. (n = 6) and one-way ANOVA followed by Tukey’s multiple range test. ^*^p < 0.001 as compared to saline control group,^ #&^*p* < 0.001 as compared to OVA group and ^&^*p* < 0.05 as compared to OVAA+A group

Parameters	Salin control	OVA	OVAA	OVAA+A	OVA+vehicle
Total WBC count/ml	4922 ± 123.02	8015 ± 178.84*	5118 ± 195.74^#.&^	7185 ± 250.14	7874 ± 301.25
Lymphocyte (%)	50.37 ± 0.89	24.84 ± 1.63*	48.33 ± 1.14^#.&^	26.41 ± 2.04	25.92 ± 1.46
Eosinophil (%)	10.64 ± 0.76	24.38 ± 0.40*	11.38 ± 0.38^#.&^	25.39 ± 2.44	25.12 ± 1.42
Monocyte (%)	20.40 ± 0.36	29.29 ± 0.47*	20.93 ± 0.47^#.&^	29.34 ± 1.45	30.10 ± 2.04
Neutrophil (%)	18.60 ± 1.48	21.49 ± 1.86*	19.36 ± 1.11^#.&^	22.87 ± 2.47	21.21 ± 1.35

*Effect of CB2 agonist treatment on total protein level*

## Discussion

Asthma is an immuno-inflammatory disease characterized by inflammation of the bronchi, and hypersensitivity of the airway. Inflammatory cells in the asthma, especially mast cells, neutrophils, eosinophils, and basophils inflammation of the area passes into the IgE and various cytokine production causes (35). Therefore, anti-inflammatory treatment, β2 adrenergic receptor agonists, and phosphodiesterase enzyme inhibitors are used in the treatment of asthma (36–38). However, as seen in recent studies (39–41), their inadequacy or side effects have become controversial. In addition, many medications have been tried and tried to improve asthma (7, 23, 27, 30, 32, 40, 42). OVA is known to be an important agent in the development of bronchial-asthma model in the rats, by increasing the levels of cytokine, IgE, MDA in BALF and serum, and by the migration of inflammatory cells into areas of inflammation (27, 32). As a result, inflammatory mediators such as TNF-α and ILs increase and cause bronchoconstriction (43). 

In the present study, it was investigated if CB2 synthetic derivative AM1241 had a protective effect on OVA-induced asthma in rats. Anti-inflammatory effect of the known CB2 receptor agonist (10, 25, 44) by the secretion of cytokine via its own CB2 receptors on inflammatory cells (45). As in the previous studies (46), in the OVA-induced asthma model of rat, it was found that AM1241 reduced weight loss and lung/body weight ratio, PFTs (pulmonary function tests), blood pressure, total WBC count, the number of immune cells such as neutrophil, monocyte, and eosinophil. It has been found that it decreases IgE, TNF-α, MDA levels and significantly increases GSH levels. In a study by Vuolo *et al*. (2), it was found that cannabinoids reduce cytokine levels in rats in the asthma model, but no evidence has been found for the effect of cannabinoids on cytokine levels via CB2 receptor. Hence, in this study we investigated whether cannabinoids with an anti-inflammatory effect were able to make this effect through their receptors. In the previous study (47), it was found that OVA inhibited weight gain in rats. In the present study, the body weight of the saline control group on the 22^th^ day was 327 ± 5.85 g (31% increase) and this value was found to be 189 ± 5.77 g (24% decrease) in the OVA group. In the OVAA group, body weight was found to be 296 ± 11.23 g (19% increase) while body weight was 205 ± 10.34 g (18% decrease) in OVAA + A group. A 37% difference was found between the agonist group and the antagonist group. This may explain to us that the cannabinoids prevent body weight loss through their own receptors. That is, the effect of AM1241 is understood to be via the CB2 receptor. Previous studies have supported the results of our study (44, 48–50). Previous studies show that OVA modifies PFTs (27, 51). Thus, PFTs are important indicators to measure the size of lung injury and to know whether the CB2 agonist AM1241 has a protective effect. In our study, Peak inspiratory flow (PIF), Peak expiratory flow (PEF), expiratory volume (EV), tidal volume (TV), the frequency of breathing (f), and enhanced pause (P_enh_) value were measured. In addition, these parameters were also used as a distinctive marker of acute lung inflammation (52). PFTs results of the previous studies of OVA-induced asthma model support the results of our study (53). However, CB2 agonist administration resulted in improvement of these parameters while CB2 antagonist inhibited this amelioration.

Collins *et al*. (54) demonstrated that because of impaired lung function, haemoglobin has been suggested to be saturated with oxygen saturation (54). Hereby, the pulmonary test is an important measurement of the degree to which the gas exchange takes place in the lung. Because lung function is impaired by asthma, the body will increase systolic and diastolic blood pressures and heart rate to close the oxygen gap. In this study, the increase in systolic and diastolic blood pressure in the OVA group supports this hypothesis. In the OVA group, systolic blood pressure increased by 61.73% was compared to the saline control group but it was found to decrease by 39.63% in the CB2 agonist group compared to the OVA group, as consistent with previous ﬁndings (55). In the antagonist group, it was found to increase by 32.03% compared to the agonist group. In a study conducted by Mukherjee *et al*. (2017), systolic and diastolic blood pressure increased in the OVA group (27).

Macrophages and monocytes are known to be essential for the initiation of the immune response. Macrophages are basically cells that are responsible for fighting allergens and destroying them (56). When doing this task, the macrophages are either intended to heal or to destroy the tissue (57). Macrophages initiate tissue repair when IL-4 level increases (58, 59). As in the previous study, our study suggested that there were changes in cell count in the BALF of OVA-induced rats (2, 60). Namely; CB2 agonist administration reduced total WBC in BALF. Both CB2 agonist and antagonist administration did not decrease total WBC count in BALF.

Inflammatory cells in the immune response together with some plasma proteins leakage out of the blood vessel (7). The amount of this microvascular leak increases in proportion to the severity of inflammation. The leakage of plasma proteins into both the inflamed tissue and the alveolar space depends on the loosening of the vascular endothelium.

Consistent with the previous ﬁndings, the results of the present investigation also showed that in OVA-induced rats, total protein content in lung tissue and BALF increased compared to the saline control group, whereas CB2 agonist administration reduced total protein content in both BALF and lung tissue (61). Eosinophils are known to be important in both innate and acquired immunity (62). In our study, as in the previous studies, both BALF and serum have been shown to increase the number of eosinophils as the lungs were induced by OVA (63). The accumulation of eosinophils has been shown to be associated with increased IL-4, IgE, and TNF-α production (2, 29, 64). In our study, CB2 agonist administration in OVA-induced asthma was found to reduce the accumulation of eosinophils, IL-4, IgE and TNF-α, although CB2 antagonist application found no reduction in the amount of these substances. As shown in previous studies (2), OVA application increased the level of IL-4, IgE, and TNF-α in BALF, serum, and lung homogenate.

It has been shown in the studies conducted by Arslan *et al*. that MDA level is an indicator of oxidative stress (42). In our study, it was found that the application of OVA significantly increased MDA level but CB2 agonist application decreased MDA level. CB2 agonist administration resulted in a significant increase in GSH content in the lung homogenate compared to the OVA group.

## Conclusion

The results of our study suggest that CB2 agonist administration to OVA-induced asthmatic rats exerts anti-asthmatic potential through inhibition of parameters such as IgE, IL-4, TNF-α, microvascular escape, and oxidative stress. These findings may be a new treatment of asthma by the anti-oxidant activity of the CB2 agonist known for its anti-inflammatory effect.
